# Digital Frequency-Domain MIMO Equalizer Enabling Six-LP-Mode Strong-Coupling IM/DD MDM Optical Transmission System

**DOI:** 10.3390/s25082562

**Published:** 2025-04-18

**Authors:** Jianyu Long, Chen Wang, Ying Wu, Bohan Sang, Chengzhen Bian, Xiongwei Yang, Long Zhang, Yifan Chen, Qinyi Zhang, Ying Wang, Yichen Li, Wen Zhou, Kaihui Wang, Bo Liu, Lei Shen, Jianjun Yu

**Affiliations:** 1 State Key Laboratory of ASIC and System, Key Laboratory for Information Science of Electromagnetic Waves (MoE), School of Information Science and Technology, Fudan University, Shanghai 200433, China; jylong22@m.fudan.edu.cn (J.L.); 20210720078@fudan.edu.cn (C.W.); yingwu23@m.fudan.edu.cn (Y.W.); bhsang20@fudan.edu.cn (B.S.); 24110720124@m.fudan.edu.cn (C.B.); xwyang22@m.fudan.edu.cn (X.Y.); longzhang21@m.fudan.edu.cn (L.Z.); chenyf327@mail2.sysu.edu.cn (Y.C.); qinyizhang23@m.fudan.edu.cn (Q.Z.); yingwang24@m.fudan.edu.cn (Y.W.); 23210720197@m.fudan.edu.cn (Y.L.); zwen@fudan.edu.cn (W.Z.); khwang@fudan.edu.cn (K.W.); bo@nuist.edu.cn (B.L.); 2 Institute of Optics and Electronics, Nanjing University of Information Science and Technology, Nanjing 210044, China; 3 Yangtze Optical Fiber and Cable Joint Stock Limited Company (YOFC), Wuhan 430073, China; shenlei@yofc.com

**Keywords:** digital signal processing, intensity modulation and direct detection, mode division multiplexing, multiple-input–multiple-output equalizer

## Abstract

Mode division multiplexing (MDM) techniques provide significant enhancement of the capacity of optical intensity modulation and direct detection (IM/DD) short-reach communication systems, like the datacenter interconnection scenarios. While the introduction of multiple modes leads to mode coupling that will extremely deteriorate the received signals, two approaches have been explored to address this issue: one involves the application of all-link weakly coupled components to suppress modal crosstalk, while the other utilizes optical multiple-input–multiple-output (MIMO) equalizers based on optical devices for signal decoupling. However, pure digital signal processing (DSP)-based schemes for mode decoupling in IM/DD MDM systems with strong mode coupling remain unexplored. In this paper, we propose to use a frequency-domain MIMO equalizer for compensating the modal interference in the strong-coupling linear-polarized (LP) MDM IM/DD system. The signal recovery capability of the proposed method is verified through numerical simulation. Finally, we successfully experimentally demonstrate the transmission of on–off-key (OOK) signals in a six-LP-mode strong-coupling MDM IM/DD system over a 10 km few-mode fiber, employing a pair of strong-coupling mode multiplexers/demultiplexers. The experimental results indicate that, with the frequency-domain MIMO equalizer, OOK signals from all modes can be recovered with an 11% hard-decision forward error correction threshold of 8.3 × $10^{-3}$. The proposed method facilitated by flexible DSP software offers an alternative for short-reach communication systems and has the potential to advance the practical application of MDM techniques in the future.

## 1. Introduction

Mode division multiplexing (MDM) technology has recently gained significant attention from researchers, emerging as a promising strategy to enhance the capacity of optical fiber communication systems [1,2]. Diverging from the wavelength division multiplexing (WDM) technology, few-mode fiber (FMF) exploits modes as spatial channels, thereby amplifying the transmission capacity manifold in comparison to conventional single-mode fiber (SMF)-based systems [3]. Numerous long-haul MDM systems incorporating coherent detection and multi-input–multi-output (MIMO) digital signal processing (DSP) have been reported [4,5]. Nonetheless, in short-reach scenarios such as datacenter interconnections (DCIs), coherent detection schemes prove not only economically burdensome for transceivers but also intricate, while intensity modulation and direct detection (IM/DD) schemes, in contrast, emerge as more pragmatic [6].

In MDM IM/DD systems, modal coupling inevitably accumulates during FMF transmission, causing inter-mode crosstalk that renders signals unrecoverable. Two primary approaches have been developed to address this challenge. The first employs a weakly coupled architecture requiring the stringent suppression of modal crosstalk throughout the entire MDM link, including mode multiplexers/demultiplexers (MMUX/MDEMUX), FMF components, and interconnections. Numerous studies [7,8,9,10,11,12,13] have demonstrated this approach. For example, Ref. [14] achieved 48 Gbps DD-OFDM transmission over a 100 m FMF using two vector modes with >12.5 dB isolation without MIMO DSP. Similarly, Ref. [15] demonstrated 152 Gbps MIMO-free PAM-4 transmission using OAM mode groups over a 5 km FMF with >21 dB isolation. Another notable example [16] realized 1.84 Tbps PAM-6 transmission in a weakly coupled six-LP-mode MDM-WDM system over a 20 m OM2 fiber. However, maintaining such high isolation levels across all link components proves challenging in practice [17,18,19,20,21], as fabrication tolerances, splicing, and field deployment may convert weak coupling into strong coupling, reviving the modal crosstalk [22,23].

The second explored route for MDM IM/DD systems is the optical MIMO scheme [24]. Through the utilization of an optical MIMO pre-coder at the transmitter side, channel estimation obtained from a training sequence can be loaded, resulting in signal crosstalk suppression. Ref. [24] initially proposed this, along with two other optical MIMO schemes in the MDM system, demonstrating their feasibility through simulation. Subsequently, in Ref. [25], the researchers delved further into optical convex optimization-based phase retrieval MIMO schemes with a low-complexity implementation, employing an array of Mach–Zehnder interferometers for optical MIMO processing. Numerical simulation results substantiated their feasibility. In Ref. [26], the researchers introduced a self-configuration method for the optical MIMO unit to track channel dynamics. The use of multiple in-mesh p-i-n and avalanche photodetectors, along with Mach–Zehnder interferometers, enables signal recovery. Although the optical MIMO scheme offers a reliable solution for mode coupling, its implementation, involving multiple photodetectors and Mach–Zehnder interferometers, is inherently complex.

Although optical MIMO schemes and weakly coupled systems have been explored as potential solutions for MDM IM/DD systems, they come with significant limitations. Optical MIMO schemes, while effective in mitigating mode coupling, require complex implementations involving multiple photodetectors and Mach–Zehnder interferometers, leading to higher system complexity and cost. On the other hand, weakly coupled systems rely on the stringent suppression of modal crosstalk throughout the entire transmission link, which is challenging to maintain in practical scenarios. Factors such as fiber fabrication imperfections, splicing, and field deployment can easily transform weak coupling into strong coupling, reintroducing modal crosstalk and degrading system performance. In contrast, the proposed electrical-domain MIMO-DSP scheme offers a more practical and flexible solution, capable of compensating for strong mode coupling without the need for complex optical components or strict isolation requirements [27,28,29,30]. For the electrical MIMO-DSP scheme in the MDM system for decoupling signals, many works based on coherent detection are reported [31,32], including some of our groups [4,5,33] and some other works with narrow-band LDPC codes [34,35,36], while the literature based on IM/DD systems is rather sparse [37].

In this paper, we propose the utilization of electrical-domain MIMO-DSP to compensate for modal interference in strong-coupling LP MDM IM/DD systems. The theoretical feasibility of digital MIMO-DSP used in the MDM strong-coupling IM/DD transmission system, without relying on any optical MIMO, is presented to some extent and experimentally demonstrated. In contrast to weakly coupled or optical MIMO-mandatory schemes, the proof indicates that electrical-domain MIMO-DSP can recover transmitted information when the DD scheme on the receiver side can only obtain the intensity of the signal without the phase. Through numerical simulations of strongly coupled IM/DD systems, we verify the capability of electrical-domain MIMO-DSP to recover signals impaired by strong mode coupling. Finally, an experiment is conducted, and the results from our six-LP-mode strong-coupling MDM IM/DD system show that, with digital MIMO-DSPs, up to 36 GBaud OOK signals received from all modes can be recovered to satisfy the 11% hard-decision forward error correction (HD-FEC) requirement. The proposed electrical-domain MIMO-DSP is shown to be feasible from theoretical, simulation, and experimental perspectives.

The main contributions of this article are listed as follows:

1 We propose and experimentally validate the first pure digital frequency-domain MIMO equalizer for strong-coupling MDM IM/DD systems, providing a practical alternative to complex optical MIMO implementations and overcoming the limitations of weakly coupled architectures.
2 We establish the theoretical foundation for electrical-domain DSP in strong-coupling scenarios through comprehensive analysis and numerical simulations, including the critical relationship between equalizer taps and mode coupling characteristics.
3 We demonstrate a 216 Gbit/s (194.6 Gbit/s net) transmission in a six-LP-mode strong-coupling IM/DD system using pure DSP schemes, achieving successful recovery of all modes below the HD-FEC threshold.

The remainder of this paper is organized as follows. Section 2 presents the principles of IM/DD MDM systems and details the frequency-domain MIMO equalizer design. Section 3 describes the simulation setup and results validating the equalizer’s performance under various coupling conditions. Section 4 details the experimental setup for the six-LP-mode transmission system. Section 5 presents and analyzes the experimental results, including comparisons of different MIMO implementations. Finally, Section 6 concludes this paper and discusses future research directions.

## 2. Principles

### 2.1. Intensity Modulation

Consider an IM/DD system. On the transmitter side, for the simplest case, suppose a signal to be transmitted s(t) is an on–off-key (OOK) signal and is normalized to [−1,1], namely,

(1) s(t)=±1

It is sent to a Mach–Zehnder Modulator (MZM). Suppose the MZM is correctly biased in the quadrature point, then the modulation voltage can be divided into a constant bias ($u_{B}$) and a time-varying modulation ($u_{m}\left(t\right)$), which can be described by

(2) $$\begin{array}{c} u\left(t\right)=u_{B}+u_{m}\left(t\right)=-\frac{1}{2}V_{\pi }+\alpha \cdot \frac{s(t)}{\left|\max(s(t))-\min(s(t))\right|}V_{\pi }=\frac{1}{2}\left(\alpha s\left(t\right)-1\right)V_{\pi } \end{array}$$

where $V_{\pi }$ is the voltage needed to induce a π phase shift between the arms, and α is the normalized amplitude of the driving voltage. Suppose the input optical carrier is $E_{\mathrm{in}}=e^{j\nu t}$; therefore, the optical modulated signal can be expressed as

(3) $$\begin{array}{c} E_{\mathrm{out}}\left(t\right)=E_{\mathrm{in}}\cos\left(\frac{\pi }{2V_{\pi }}u\left(t\right)\right)=e^{j\nu t}\cos\left[\frac{\pi }{4}\left(\alpha s\left(t\right)-1\right)\right] \end{array}$$

Accordingly, the output optical power can be expressed as follows:

(4) $$\begin{array}{c} P_{\mathrm{out}}\left(t\right)=\frac{1}{2}P_{\mathrm{in}}\left[1+\sin\frac{\alpha s(t)\pi }{2}\right]=\frac{1}{2}\left[1+\sin\frac{\alpha s(t)\pi }{2}\right] \end{array}$$

If we restrict the operating range of the MZM to the linear region, and equally limit the α to a small range, e.g., α≤0.5, we have

(5) $$\begin{array}{c} P_{\mathrm{out}}\left(t\right)=\frac{1}{2}\left[1+\alpha s\left(t\right)\right]. \end{array}$$

And the modulated optical signal is

(6) $$\begin{array}{c} E_{\mathrm{out}}\left(t\right)=e^{j\nu t}\frac{\alpha }{2}s\left(t\right). \end{array}$$

Once we have the expression of the modulated signal in Equation (6), we can consider its recovery on the receiver side.

### 2.2. PAM Signals in IM/DD MDM Systems

**Figure 1:**
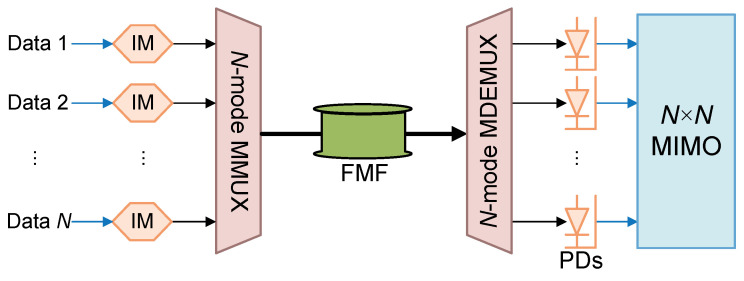
A typical MDM IM/DD system with *N* modes for data carrying. *N* lanes data are modulated by intensity modulators (IMs), aggregated by MMUX, transmitted via FMF, and split by MDEMUX. Intensity of each lane is detected by photodetector (PD) and sent into digital MIMO DSP.

Consider an MDM IM/DD system with *N* modes carrying *N* independent signals, shown in Figure 1. Denote

(7) $$\begin{array}{cc} s_{N\times 1} & ={\left[s_{1}\left(t\right),s_{2}\left(t\right),\ldots ,s_{N}\left(t\right)\right]}^{T}\\ u_{N\times 1} & ={\left[E_{{\mathrm{out}}_{1}}\left(t\right),E_{{\mathrm{out}}_{2}}\left(t\right),\ldots ,E_{{\mathrm{out}}_{N}}\left(t\right)\right]}^{T}\\ p_{N\times 1} & ={\left[P_{{\mathrm{out}}_{1}}\left(t\right),P_{{\mathrm{out}}_{2}}\left(t\right),\ldots ,P_{{\mathrm{out}}_{N}}\left(t\right)\right]}^{T}. \end{array}$$

From Equations (5) and (6), we have

(8) $$\begin{array}{cc} \; & u=\frac{1}{2}e^{j\nu t}\alpha s\\ \; & p=\frac{1}{2}\left[1_{N\times 1}+\alpha s\right]. \end{array}$$

Let us denote H as the channel matrix and y as each signal output from MDEMUX. There holds the relationship

(9) $$\begin{array}{c} y=Hu \end{array}$$

where H is a complex, invertible, and objectively existing matrix [38,39,40]. In coherent detection, where the intensity and phase of each mode can be achieved, once the training sequences are sent, the channel matrix H can be estimated by MIMO-DSPs.

However, on the receiver side, there are *N* photodetectors for obtaining signals from MDEMUX, and only intensity d can be achieved. Ignoring the additive noise, we have

(10) $$\begin{array}{c} d_{i}={\left|u_{i}\right|}^{2},\;i=1,2,\ldots ,N \end{array}$$

One should note that on the transmitter side, we directly modulate the optical power p. Similarly, if we define a matrix R as the intensity counterpart of the channel matrix, describing the relationship between the optical power at the transmitter side and receiver side, we shall have

(11) $$\begin{array}{c} d=Rp \end{array}$$

d and p are all real vectors, standing for the intensity of the optical signals at the receiver or transmitter. R conveys the relationship between these two real-value vectors, so that R cannot be a complex matrix. What we should do is use MIMO-DSPs to recover R. It is noteworthy that the matrix recovered using MIMO-DSP in this context is not equivalent to the coherent system’s channel matrix H, which typically includes both amplitude and phase information. Instead, it corresponds to a power-related matrix R analogous to the channel matrix.

Therefore, Equation (11) signifies that by establishing the relationship R between the transmitted power p and the received power d, we can recover p from d. In other words, the use of electrical-domain MIMO-DSP on d is required to reconstruct the matrix R, subsequently enabling the retrieval of the transmitted signal p. In summary, this to some extent demonstrates the feasibility of signal recovery in IM/DD strong mode coupling MDM systems through the application of electrical-domain MIMO-DSP.

### 2.3. Time-Domain MIMO Equalizer

We start from the time-domain MIMO equalizer. Denote $d_{i,k}={\left[d_{i,k-\frac{L-1}{2}},d_{i,k-\frac{L-1}{2}+1},\ldots ,d_{i,k+\frac{L-1}{2}}\right]}^{T}$ as the *k*th received signal for the *i*th mode, where *L* is the equalizer length. The real-valued $LN^{2}$ equalizer matrix R is defined as

(12) $$\begin{array}{c} R=\left[R_{i,j}\right] \end{array}$$

where *L*-length $R_{i,j}$ denotes the (i,j)-th column vector of R. The *k*th symbol of the equalizer output for the *i*th mode $x_{i,k}$ can be obtained as

(13) $$\begin{array}{c} x_{i,k}=\sum_{j=1}^{N}{r_{i,j}}^{T}d_{i,k} \end{array}$$

Employing the data-aided or decision-directed method can update the equalizer. For example, using the least mean square (LMS) method, the *k*th desired standard output symbol for the *i*th mode is $z_{i,k}$, and the error signal $e_{i,k}$ can be calculated as

(14) $$\begin{array}{c} e_{i,k}=z_{i,k}-x_{i,k} \end{array}$$

As the updating principle, the corresponding formula with the step-size parameter μ is derived as

(15) $$\begin{array}{c} r_{i,j}\leftarrow r_{i,j}+\mu e_{i,k}d_{j,k} \end{array}$$

As can be seen, the time-domain MIMO equalizer outputs only one symbol at a time and updates once per symbol. While this approach may enhance accuracy, it also introduces higher computational overhead.

### 2.4. Frequency-Domain MIMO Equalizer

Compared with the time-domain MIMO equalizer, the frequency-domain MIMO equalizer divides the input signals into several parts. The input sequence undergoes a series-parallel transformation, breaking it into data chunks of specific lengths, $L_{1}$. These data chunks are then fed into the equalizers with a length of *L*. The frequency-domain MIMO equalizer matrix $R_{f}$ is defined as

(16) $$\begin{array}{c} R^{f}=\left[\mathrm{FFT}\left(r_{i,j}^{f};0_{L}\right)\right] \end{array}$$

FFT (Fast Fourier Transform) and IFFT (Inverse Fast Fourier Transform) enable the efficient implementation of linear convolution and linear correlation. Simultaneously, it utilizes the overlap-save method and the overlap-add method for computing linear convolution. In the computational process, efficiency is maximized when employing a 50% overlap. Hence, a 50% overlap rate is adopted here. In the algorithm, when the block length $L_{1}$ is equal to the filter length *L*, the computation complexity is minimized. Therefore, in the frequency-domain LMS adaptive equalization, the filter length is set as $L=L_{1}$. Following the overlap-save method, zero-padding is applied to the filter, with the padding length equal to the block length *L*.

Denote $d_{i,K}={\left[d_{i,KL+1},d_{i,KL+2},\ldots ,d_{i,KL+(L-1)}\right]}^{T}$ as the *K*th received signal block for the ith mode. Then, denote $D_{i,K}=\mathrm{FFT}\left([d_{i,K-1};d_{i,K}]\right)$ as the Kth frequency-domain-received signal block for the ith mode.

Employing the overlap-save method, due to the circular nature of the convolution result in the computed output, only the last *L* elements are retained. The *K*th *L*-length of the equalizer output block for the *i*th mode $x_{i,K}$ can be obtained as

(17) $$\begin{array}{c} x_{i,K}=\left[\sum_{j=1}^{N}\mathrm{IFFT}\left(R_{i,j}^{f}⊙D_{i,K}\right)\right] \end{array}$$

with the last *L* elements.

Here, the ⊙ denotes the Hadamard-product, meaning the element-wise multiplication.

Similarly, employing the data-aided or decision-directed method can update the equalizer. For example, using the LMS method, the *K*th desired standard output symbol block for the *i*th mode is $z_{i,K}$, and the error signal $e_{i,K}$ can be calculated as

(18) $$\begin{array}{c} e_{i,K}=z_{i,K}-x_{i,K} \end{array}$$

Since the first *L* elements in Equation (17) are discarded, the frequency-domain error signal block $E_{i,K}$ needs to have the first *L* data points set to zero to facilitate the transformation to the frequency domain.

(19) $$\begin{array}{c} E_{i,K}=\mathrm{FFT}\left(\left[0_{L};e_{i,K}\right]\right) \end{array}$$

As the updating principle, the corresponding formula with the step-size parameter μ is derived as

(20) $$\begin{array}{c} {\mathrm{EX}}_{i,j,K}=\mathrm{IFFT}\left(\left[E_{i}⊙X_{j}\right]\right) \end{array}$$

with the first *L* elements.

(21) $$\begin{array}{c} R_{i,j}^{f}\leftarrow R_{i,j}^{f}+\mu \mathrm{FFT}\left(\left[EX_{i,j,K};0_{L}\right]\right) \end{array}$$

where in Equation (17), the first *L* elements are discarded, while in Equation (20), the last *L* elements are omitted. Therefore, the update of the weight coefficients in the frequency-domain LMS algorithm is as above.

The block-wise updated frequency-domain MIMO equalizer exploits the relationship between frequency-domain multiplication and time-domain convolution, resulting in a significant reduction in computational complexity. However, it is important to note that block-wise updating may potentially lead to a decrease in the accuracy of the equalizer.

### 2.5. T/2-Spaced Frequency-Domain MIMO Equalizer

Similar to the frequency-domain MIMO equalizer, the T/2 frequency-domain MIMO equalizer requires a signal sampled at twice the rate. On the signal input side, the signal is split into odd and even sequences. Likely, we have $d_{i,K,o}$ and $d_{i,K,e}$ and the corresponding $D_{i,K,o}$ and $D_{i,K,e}$.

Additionally, two sets of MIMO equalizers with identical structures are needed to separately equalize the odd and even sequences. Like Equation (16), we have

(22) $$\begin{array}{c} \begin{array}{cc} & R_{o}^{f}=\left[\mathrm{FFT}\left(r_{i,j,o}^{f};0_{L}\right)\right]\\ & R_{e}^{f}=\left[\mathrm{FFT}\left(r_{i,j,e}^{f};0_{L}\right)\right] \end{array} \end{array}$$

At the signal output side, there are slight differences from Equation (17). The results of the corresponding MIMO equalizers for the odd and even sequences are directly summed to obtain the signal output.

(23) $$\begin{array}{c} x_{i,K}=\left\{\sum_{j=1}^{N}\left[\mathrm{IFFT}\left(R_{i,j,o}^{f}⊙D_{i,K,o}\right)+\mathrm{IFFT}\left(R_{i,j,e}^{f}⊙D_{i,K,e}\right)\right]\right\} \end{array}$$

with the last *L* elements.

Finally, similar to Equations (18)–(20), using the output signal, errors for the two MIMO equalizers are the same and calculated, and updates are made independently. For instance, using methods like LMS, the updating principles are similar to Equation (21)

(24) $$\begin{array}{c} \begin{array}{cc} & R_{i,j,o}^{f}\leftarrow R_{i,j,o}^{f}+\mu \mathrm{FFT}\left(\left[EX_{i,j,K,o};0_{L}\right]\right)\\ & R_{i,j,e}^{f}\leftarrow R_{i,j,e}^{f}+\mu \mathrm{FFT}\left(\left[EX_{i,j,K,e};0_{L}\right]\right) \end{array} \end{array}$$

The T/2 frequency-domain MIMO equalizer processes two samples per symbol (2 Sa/Sym) signal sequences and outputs at a rate of 1 Sa/Sym. This configuration results in improved signal recovery performance compared to its T/1 counterpart. In summary, the principles of the T/1-spaced time-domain MIMO equalizer are relatively straightforward. But, in the time domain, the filter tap coefficients need to be updated and a symbol output is generated for each processed symbol. While this approach allows for the consideration of errors for each symbol, with feedback updates to the filter taps, it introduces a significant computational burden, resulting in a notable reduction in processing speed. On the other hand, the T/1-spaced frequency-domain MIMO equalizer leverages FFT and IFFT and employs an overlap-save method. This technique, involving the multiplication of a data block in the frequency domain with filter taps, enables the rapid computation of the convolution of the corresponding data block in the time domain with the filter. This substantially reduces the computational workload and enhances the processing speed. However, the grouped updating method in the T/1-spaced frequency-domain MIMO equalizer results in the error updates for filter taps at specific positions being calculated only from input signals at fixed intervals. This may lead to a compromise in the performance of the equalizer. Given that sampling from the ADC often exceeds 1 Sa/Sym, utilizing this additional sampling may contribute to signal recovery gains. Hence, the T/2-spaced frequency-domain MIMO equalizer has emerged. This equalizer balances odd and even sequences of signals using 2 Sa/Sym, and compared to the T/1 equalizer, it may mitigate the impact of clock errors, resampling, and deviations between signal points and sampling points to some extent. Therefore, in pursuit of higher computational efficiency and improved signal recovery, we opt to utilize the T/2-spaced frequency-domain MIMO equalizer in this context.

## 3. Simulation Setup and Results

**Figure 2:**
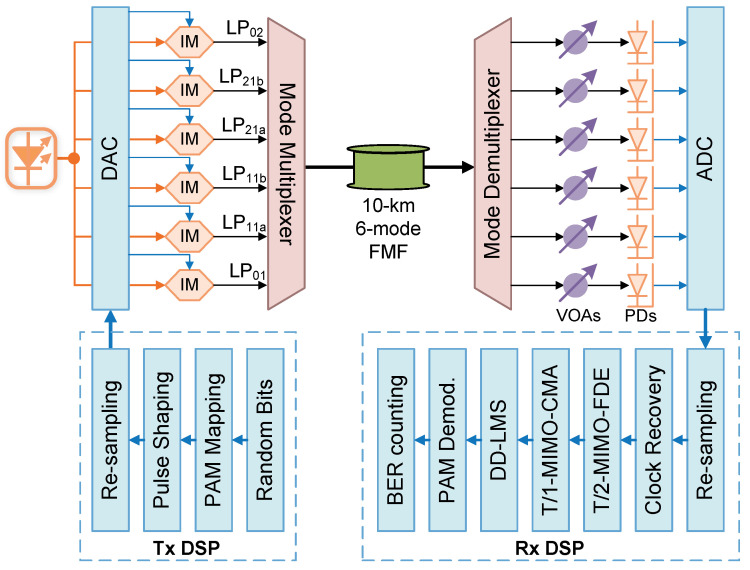
Simulation setup of the IM/DD strong-coupling MDM transmission system with 6 modes for data carrying.

To verify the feasibility of the proposed electrical-domain MIMO-DSP for the IM/DD strong-coupling MDM system, we conduct a numerical simulation study. Figure 2 illustrates the simulation setup, conducted by using a commercial optical simulation software. The 36 GBaud OOK signals are initially generated by 6 independent random-bit streams and sent for OOK mapping. A pulse-shaping process includes up-sampling to 2 Sa/Sym and passing through a root-raised cosine filter with a roll-off factor of 0.01. The 6-lane optical signals are generated by 6 identical Mach–Zehnder Intensity Modulators and transmitted to the MMUX serving as the signals for the 6 modes. After 10 km 6-mode strong-coupling FMF transmission, the signals from the 6 modes can be obtained by an MDEMUX, attenuated by VOAs, and detected by photodetectors. DAC can capture the 6-lane signals for DSP by an offline MATLAB 2021b program. After resampling to 2 Sa/Sym and clock recovery, the 6-lane signals are processed by T/2-MIMO-FDE, T/1-MIMO-CMA, and independent DD-LMS. After OOK demodulation, the BER can be measured by error counting. In the simulation, the model “LaserCW”, “SplitterPow_1_N”, “ModulatorDiffMZ_DSM”, “CombinerSplitterMM”, “FiberMM”, “CombinerSplitterMM”, “Attenuator”, and “Photodiode” are used to emulate the optical source, optical coupler, Mach–Zehnder Intensity Modulator, MMUX, FMF, MDEMUX, VOA, and photodetector, respectively, where the MMUX, FMF, and MDEMUX are dependent on the module “SolverFiberMeasuredMM”. The “LaserDriver”, “RiseTimeAdjust”, and “DAC” are used to emulate the amplified signals loading to DACs. The “ADC” module is for emulating the ADC. The parameters for these key devices in the simulation are outlined in Table 1 and Table 2. It should be noted that in the simulation parameter list, the parameters named “Mode Coupling”, “Mode Grouping”, and “All Modes Correlation Length” should be set to “Yes”, “All Modes”, and “10 km”, respectively, so that strong mode coupling can be correctly simulated within all modes and the whole FMF link.

**Figure 3:**
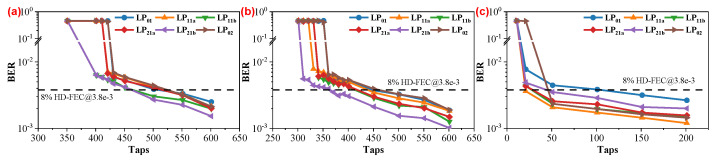
BER performance of 36 GBaud OOK signals versus the taps of the T/2−MIMO−FDE. All mode coupling with an “All Modes Correlation Length” of (**a**) 10 km; (**b**) 5 km; and (**c**) 0 m with optical back-to-back case.

In Figure 3, we demonstrate the relationship between the BER performance and the taps of the T/2-MIMO-FDE, to illustrate whether the electrical-domain MIMO-DSP can recover the signals from the strong-coupling MDM link with the DD scheme. To qualitatively assess the capability of electrical-domain MIMO-DSP in mitigating strong mode coupling, we varied a critical parameter, namely, the “All Modes Correlation Length”, which determines the extent of strong mode coupling in the simulated environment. This parameter is set to values representing the entire length of the FMF (10 km), half of the FMF length (5 km), and the 0 m optical back-to-back (OBTB) case without FMF. This adjustment aimed to scrutinize the impact of different correlation lengths on the effectiveness of electrical-domain MIMO in mitigating strong mode coupling in the simulated scenario. The three plots in Figure 3 illustrate the relationship between the number of taps and BER performance of T/2-MIMO-FDE under three different “All Modes Correlation Length” values. It is evident that, for all three scenarios, T/2-MIMO-FDE functions effectively for all mode signals when the number of taps exceeds a specific threshold. Furthermore, BER performance gradually improves with an increase in the length of taps. Conversely, when the number of taps falls below a certain threshold, T/2-MIMO-FDE fails to recover signals from certain modes until the number of taps decreases to a smaller value, eventually rendering it incapable of recovering signals from all modes. By comparing Figure 3a,b, it can be observed that the “All Modes Correlation Length” influences the number of taps in T/2-MIMO-FDE to a certain extent. According to the theoretical framework of T/2-MIMO-FDE, its taps must encompass the entire differential group delay (DGD) accumulated over the 10 km FMF time window. For a 36 GBaud signal, the minimum required number of taps can be calculated as

(25) $$\begin{array}{c} n_{\mathrm{taps}\;}\geq \frac{n_{\mathrm{DGD}}\times L_{\mathrm{FMF}}}{1/\;\mathrm{BaudRate}\;}=\frac{\left(810\times 10^{-12}\right)\times 10}{1/\left(36\times 10^{-9}\right)}=291.6 \end{array}$$

Hence, a minimum of approximately 292 taps is needed to ideally eliminate DGD. However, in the T/2-MIMO-FDE used here, it not only needs to eliminate DGD but also address strong mode coupling. Consequently, for the 5 km scenario in Figure 3b and the 10 km scenario in Figure 3a, the minimum operational tap values are approximately 361 = (292 + 69) and 431 = (292 + 139) ≈ (292 + 69 × 2), respectively. Observing that the additional taps introduced by the so-called strong mode coupling seem to be directly proportional to the length of strong coupling, it is reasonable to speculate a correlation between the number of taps in the T/2-MIMO-FDE and strong mode coupling. Considering the ability of the T/2-MIMO-FDE to reduce the BER to a threshold value, such as 3.8 $\times 10^{-3}$, when an adequate number of taps is employed, we can conclude that simulations suggest the successful application of electrical-domain MIMO in IM/DD strong mode coupling MDM transmission systems, enabling the accurate recovery of signals.

## 4. Experimental Setup

In order to further validate the feasibility of our proposed electrical-domain MIMO-DSP for IM/DD strong mode coupling MDM systems, we constructed a strong-coupling IM/DD system with six LP modes. Experimental verification is conducted to assess the validity of the proposed methodology.

**Figure 4:**
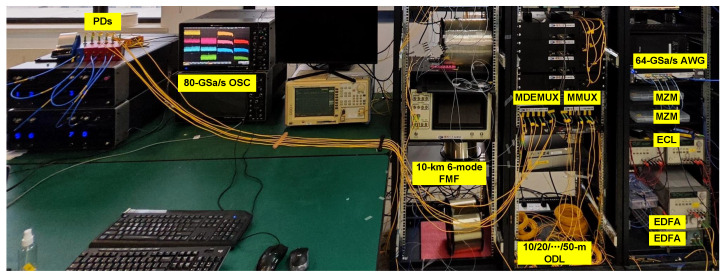
The photo of the experimental setup. The transmitter side is on the right, while the receiver side is on the left. ODL, optical delay line.

**Figure 5:**
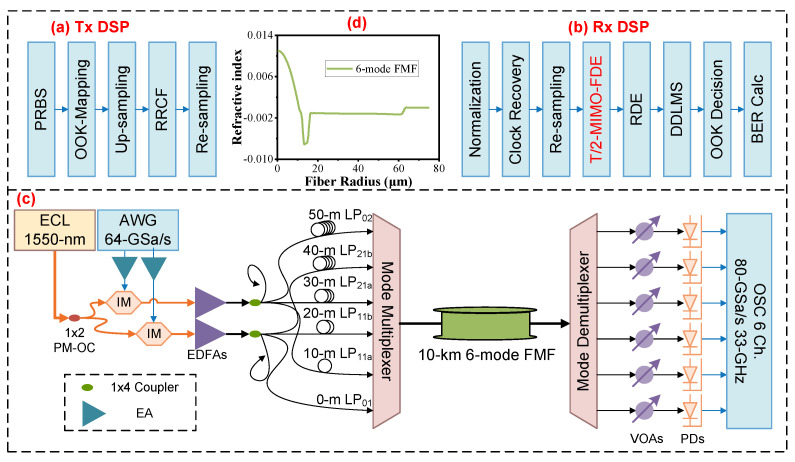
(**a**) The Tx DSP. (**b**) The Rx DSP. (**c**) The diagram of the experimental setup. ECL, external cavity laser; PM-OC, polarization-maintaining optical coupler; AWG, arbitrary waveform generator; IM, intensity modulator; EDFA, erbium-doped fiber amplifier; FMF, few-mode fiber; VOA, variable optical attenuator; PD, photodetector; OSC, oscilloscope. Inset (**d**) is the refractive index of the FMF.

Figure 4 shows the photo of the experiment. Figure 5a shows the Tx DSP of the six-LP-mode MDM IM/DD transmission system. Firstly, two pseudorandom binary sequences (PRBSs) are generated offline in binary data format. Subsequently, the sequences are mapped to OOK data. Next, in order to restrict the signal bandwidth, the OOK sequences undergo a × 2 up-sampling process to match the requirements of a root-raised cosine filter with a roll-off factor of 0.01. Finally, they are re-sampled into the specific baud rate for generating.

Figure 5c illustrates the system setup. Two independent OOK signals generated from different PRBSs with the method mentioned above are driven by an arbitrary waveform generator (AWG) whose sampling rate is up to 64 GSa/s. The two output streams from the AWG are amplified by two electrical amplifiers (EAs) and sent to two MZMs as intensity modulators with bias on the quadrature point. A 1550 nm continuous-wave light wave with an optical power of 13 dBm is generated by a free-running external cavity laser (ECL) and separated by a polarization-maintaining 1 × 2 optical coupler (PM-OC) and sent into two modulators. Two streams of modulated signals from two modulators are then sent to two identical erbium-doped fiber amplifiers (EDFAs) for amplification. Afterwards, two identical 1 × 4 optical couplers are employed to divide two optical streams into eight paths. Each 1 × 4 optical coupler selectively uses three output paths. In the first 1 × 4 optical coupler, two of the three paths undergo delays of 20 m and 40 m, respectively, while the remaining path has no delay, aimed at signal de-correlation. These three paths are then connected to an MMUX, serving as signals for the LP01, LP11b, and LP21b modes. For the three paths utilized in the second 1 × 4 optical coupler, delays of 10 m, 30 m, and 50 m are applied in comparison to the first path of the initial 1 × 4 optical coupler. Subsequently, these three paths are connected to the MMUX, serving as signals for the LP11a, LP21a, and LP02 modes. The MMUX is connected with the FMF.

The MMUX and MDEMUX in this experiment are 6-input-port all-fiber photonic lanterns, which are fabricated in the same medium as the fiber transmission and offer optimum performance in a single component, compact format. The all-fiber lantern is an adiabatic taper that provides a low-loss transition from the input fibers to the modes supported by the waveguide at its output. Due to its properties, it will definitely cause strong mode coupling and MIMO is a must for signal recovery.

Figure 5d shows the refractive index of the FMF. In addition, the loss and effective area of the 10 km FMF for LP01, LP11, LP21, and LP02 are 0.208 dB/km, 0.202 dB/km, 0.207 dB/km, and 0.215 dB/km and 90 μ$m^{2}$, 121 μ$m^{2}$, 159 μ$m^{2}$, and 161 μ$m^{2}$, respectively. The dispersion coefficient of the FMF link for LP01, LP11, LP21, and LP02 are 21.25 ps/nm/km, 21.01 ps/nm/km, 19.5 ps/nm/km, and 20.29 ps/nm/km, respectively. The differential mode delay (DMD) of the FMF for the LP01 mode to the LP11 mode, LP11 to LP21, and LP01 to LP02, is 400 ps/km, 110 ps/km, and 810 ps/km, respectively. Each output mode from a MDEMUX connected with the FMF is independently attenuated by a variable optical attenuator (VOA) and then detected by an identical photodiode (PD). The signal is captured by a 6-port oscilloscope with a sampling rate of 80 GSa/s and a bandwidth of 33 GHz.

In the offline Rx DSP (Figure 5b), each signal is firstly performed with clock recovery and then re-sampled to 2 Sa/sym. Following that, a 6 × 6 decision-directed T/2-MIMO-LMS frequency-domain equalizer (FDE) with 601 taps is applied to realize coarse equalization and 6-lane input decoupling. Before equalization, T/2-MIMO-LMS-FDE uses the first 1.5 × 10^4^ symbols in the data-aided mode for training in order to initialize the coefficients of the filter arrays. After that, 6-lane signals are sent into T/1-MIMO-CMA for further equalization. Subsequently, each data lane is processed via time-domain decision-directed LMS. A hard-decision is thereafter implemented to obtain the recovered OOK signals. Finally, the bit error rate (BER) can be further measured.

## 5. Experimental Results

**Figure 6:**
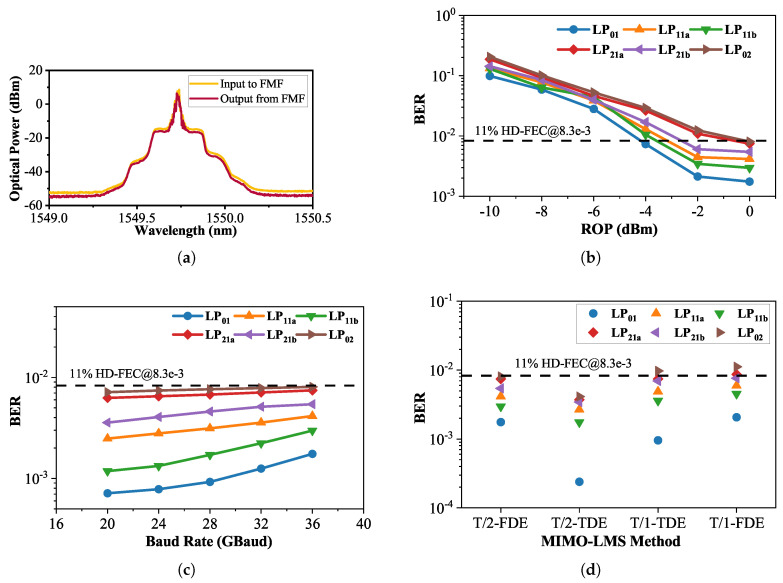
(**a**) The optical spectra of the 36 GBaud OOK signals captured at the input/output side of the 10 km FMF (resolution: 0.01 nm). (**b**) Bit error rate (BER) performance of each mode versus received optical power (ROP). (**c**) ber performance of each mode versus baud rate. (**d**) BER performance of each mode versus different MIMO-LMS methods.

We firstly transmit an OOK signal with a baud rate of 36 GBaud. The plots of the BER versus received optical power (ROP) are shown in Figure 6b, and it displays the BER performance of the 36 GBaud OOK signal for all LP modes. It is observable that the LP01 mode consistently exhibits superior BER performance compared to other modes. Additionally, within the pairs of degenerate modes, both LP11a and LP21a demonstrate a slightly worse BER compared to their respective degenerate modes. As for the higher-order mode LP02, it exhibits the poorest BER performance. Furthermore, as the ROP increases from −10 to −2 dBm, there is a noticeable improvement in the corresponding BER performance. However, when the ROP reaches 0 dBm, the change in BER performance compared to −2 dBm is relatively minor. We attribute this observation to two primary reasons. Firstly, the PD has reached its saturation in receiving optical power. Secondly, due to the necessity of utilizing a 1 × 4 OC for signal de-multiplexing, which incurs a 6 dB power loss for each output, along with losses in the MMUX and MDEMUX, such a high ROP demands elevated output power from the system’s transmitter-side EDFAs. This elevated power requirement might lead to the onset of optical fiber nonlinearity, thereby affecting the BER performance. At the ROP of −2 dBm, the BER of the recovered signals from all modes attains the 11% HD-FEC threshold of $8.3\times 10^{-3}$.

Figure 6c depicts the relationship between the BER and OOK signals under an ROP of 0 dBm. It is notable that the BER steadily increases in the range of baud rates from 20 to 36 GBaud, following a consistent trend. Moreover, at the same baud rate across different modes, the BER performance remains analogous to the previous observation based on the ROP, with LP01 exhibiting the best performance and LP02 displaying the poorest performance.

Next, to assess the performance disparities among various MIMO-LMSs, we substituted “T/2-MIMO-FDE” in Figure 5b under the conditions of 36 GBaud OOK and an ROP at 0 dBm. The substitutions were made with the T/2-spaced time-domain MIMO equalizer (T/2-MIMO-TDE), T/1-spaced time-domain MIMO equalizer (T/1-MIMO-TDE), and T/1-spaced frequency-domain MIMO equalizer (T/1-MIMO-FDE). We ensured that the tap number, μ value, and other parameters were as consistent as possible. The BER performance of each MIMO configuration was compared, as illustrated in Figure 6d.

Observations reveal that, as reasoned in Section II, the T/2-MIMO-TDE exhibits superior BER performance across all modes. This can be attributed to its practice of updating weights for each symbol, ensuring that the filter’s weights are influenced by error updates from nearly all signal positions. Furthermore, the utilization of 2 Sa/Sym preserves more sampling point information while mitigating the impact of signal points deviating from sampling points. However, it is worth noting that the execution of this program incurred significant computational time.

The BER performance of the T/2- or T/1-MIMO-FDE is consistently weaker than its time-domain counterpart, which is comprehensible. The MIMO-FDE employs a grouped calculation and updating approach, leading to error updates for filter taps at specific positions being calculated only from fixed-interval input signals. The performance gap in the filter BER may be associated with this grouping method. Nevertheless, a comparative analysis using probes during program execution indicates that time-domain equalization consumes several times, or even tens of times, the duration required for frequency-domain equalization. It is noteworthy that the T/2-MIMO-FDE exhibits superior BER performance overall. However, its signal recovery capability is weaker than the T/1-MIMO-TDE in the LP01 mode with better BER performance, while it surpasses the T/1-MIMO-TDE in the LP02 mode with poorer BER performance. The signal recovery capabilities are comparable in other modes. This is challenging to predict based on theoretical considerations. From the perspective of program execution duration, it is evident that the efficiency of the former is higher.

Finally, the superior BER performance of the T/2-MIMO-FDE over the T/1-MIMO-FDE is as expected, as elucidated in the comparison between the T/2- and T/1-MIMO-TDE. Additionally, within the range of 36 Gbaud, all modes exhibit error performance meeting the 11% HD-FEC threshold of $8.3\times 10^{-3}$. The total line bit rate is 36 × 6 = 216 Gbit/s. Taking into account an 11% overhead for HD-FEC in the 10 km 6-mode FMF IM/DD system, the net bit rate is calculated as 216/(1 + 11%) = 194.6 Gbit/s.

The experimental results validate the correct recovery of OOK signals with a data rate of up to 36 GBaud using the T/2-MIMO-FDE. Consequently, we have sufficient grounds to assert that our proposed electrical-domain MIMO scheme for IM/DD strong mode coupling MDM transmission systems is effective at the experimental level and demonstrates feasibility.

Table 3 compares the computational complexity (FLOPs/symbol) of the TDE and FDE in MIMO systems with T/2 and T/1 spacing. The FDE methods scale logarithmically with *L* ($5\log_{2}L$), while the TDE scales linearly (2L−1). T/2-MIMO requires ∼2× more FLOPs than T/1-MIMO due to higher dimensionality.

## 6. Conclusions

In conclusion, we have successfully demonstrated the feasibility of employing a frequency-domain MIMO equalizer to mitigate strong mode coupling in a six-LP-mode IM/DD MDM system, achieving the recovery of 36 GBaud OOK signals with a BER below the 11% HD-FEC threshold of $8.3\times 10^{-3}$ over a 10 km FMF. We have validated the proposed electrical-domain MIMO-DSP scheme through both simulation and experiment, showcasing its potential as a practical solution for enhancing the capacity and performance of short-reach optical communication systems. This work paves the way for the broader adoption of MDM techniques in future applications.

## Figures and Tables

**Table 1:** Parameters of the simulation.

Device	Parameter	Value
Laser	Linewidth	100 kHz
	Average Power	21 dBm
	Wavelength	1550 nm
Driver	Amplitude	1.2 V
RiseTimeAdjust	RiseTime	0.25/36 ns
MZM	Insertion Loss	6 dB
	Extinction Ratio	35 dB
DAC	Resolution	7
PD	Responsivity	1 A/W
	Thermal Noise	$10^{-12}$ A/Hz^1/2^
	Shot Noise	On

**Table 2:** Mode-relevant parameters of the simulation.

Device	Parameter	Value
Mode Solver	Supported Modes	(0, 1) (1, 1) (2, 1) (0, 2)
	Attenuation	0.208, 0.202, 0.207, 0.215 dB/km
	Relative Mode Delay	Yes
	Dispersion	21.25, 21.01, 19.5, 20.29 ps/nm/km
	DGD	0, 400, 510, 810 ps/km
	Intra-Mode-Group Delay Deviation	5–15 s/m
MMUX	Mode Solver	Mode Solver
MDEMUX	Mode Solver	Mode Solver
FMF	Mode Solver	Mode Solver
	Length	10 km
	**Mode Coupling**	**Yes**
	**Mode Grouping**	**All Modes**
	**All Modes Correlation Length**	**10 km**
	Linear Coupling—Mean Section Length	0.5 km
	Linear Coupling—Section Length Deviation	5 m

**Table 3:** Computational complexity comparison of MIMO equalization methods.

Method	FLOPs per Symbol
T/2-MIMO-FDE	$2N^{2}\left(5\log_{2}L+1\right)+1$
T/2-MIMO-TDE	$2N^{2}\left(2L-1\right)+11$
T/1-MIMO-FDE	$N^{2}\left(5\log_{2}L+1\right)$
T/1-MIMO-TDE	$N^{2}\left(2L-1\right)+5$

## Data Availability

The raw/processed data required to reproduce these findings cannot be shared at this time, as the data form part of another ongoing study.
